# Autosomal dominant congenital cataract in a Libyan Jewish family: cosegregation with a reciprocal chromosomal translocation [t(3;5)(p22.3; p15.1)]

**Published:** 2008-03-14

**Authors:** Emre Zafer, Jeanne Meck, Liora Gerrad, Elon Pras, Moshe Frydman, Orit Reish, Isaac Avni, Eran Pras

**Affiliations:** 1 Department of Obstetrics and Gynecology, Georgetown University, Washington, DC; 2 Sheba Medical Center, Danek Gartener Institute of Human Genetics, Tel Hashomer, Israel; 3 Sackler Faculty of Medicine, Tel Aviv University, Tel Aviv, Israel; 4 Department of Human Genetics, Assaf Harofeh Medical Center, Zerifin, Israel; 5 Department of Ophthalmology, Assaf Harofeh Medical Center, Zerifin, Israel

## Abstract

**Purpose:**

To describe a Jewish family of Libyan ancestry in which autosomal dominant congenital cataract segregates with an apparently balanced reciprocal chromosomal translocation.

**Methods:**

Detailed family history and clinical data were recorded. Cytogenetic studies were performed on 13 family members.

**Results:**

Embryonal cataracts cosegregated through three generations with a balanced chromosomal translocation [t(3;5)(p22.3; p15.1)] while the unbalanced translocation product, 46,XY,-5,+der(5)t(3:5)(p22:p15.1), had multiple congenital anomalies without cataracts.

**Conclusions:**

These observations suggest that an altered function of a gene at one of the translocation breakpoints on chromosome 3p22.3 or 5p15.1 is causally related to cataract development.

## Introduction

Congenital cataracts are common disorders of the eye that often cause visual impairment or blindness in children [1,2]. At least one-third of the cases are familial. Congenital cataracts are most commonly inherited as an autosomal dominant trait, but autosomal recessive and X-linked inheritance patterns have also been reported [3]. Hereditary nonsyndromic congenital cataracts are extremely heterogeneous and are usually considered to be the result of single gene mutations [1]. The presence of associated abnormalities in other organ systems marks the syndromic cataracts, which in turn can be caused by Mendelian disorders, chromosomal abnormalities, or nongenetic factors (e.g., environmental). A unique and rare subgroup among syndromic cataract is associated with chromosomal rearrangements, which are often accompanied by multiple malformations and a family history of recurrent pregnancy losses [4]. While linkage studies in families with hereditary cataracts have been the most fruitful method for the identification of cataract loci, occasionally these were identified by studying families in which cataract cosegregated with marker chromosomes [5-8]. Furthermore, defining the translocation breakpoints in these families may help in cloning the underlying causative gene [9,10], thus providing further insight into normal lens development and cataract formation.

In this report, we present a Libyan Jewish family in which congenital cataracts segregated with a balanced reciprocal chromosomal translocation t(3:5)(p22.3;p15.1).

## Methods

**Figure 1:**
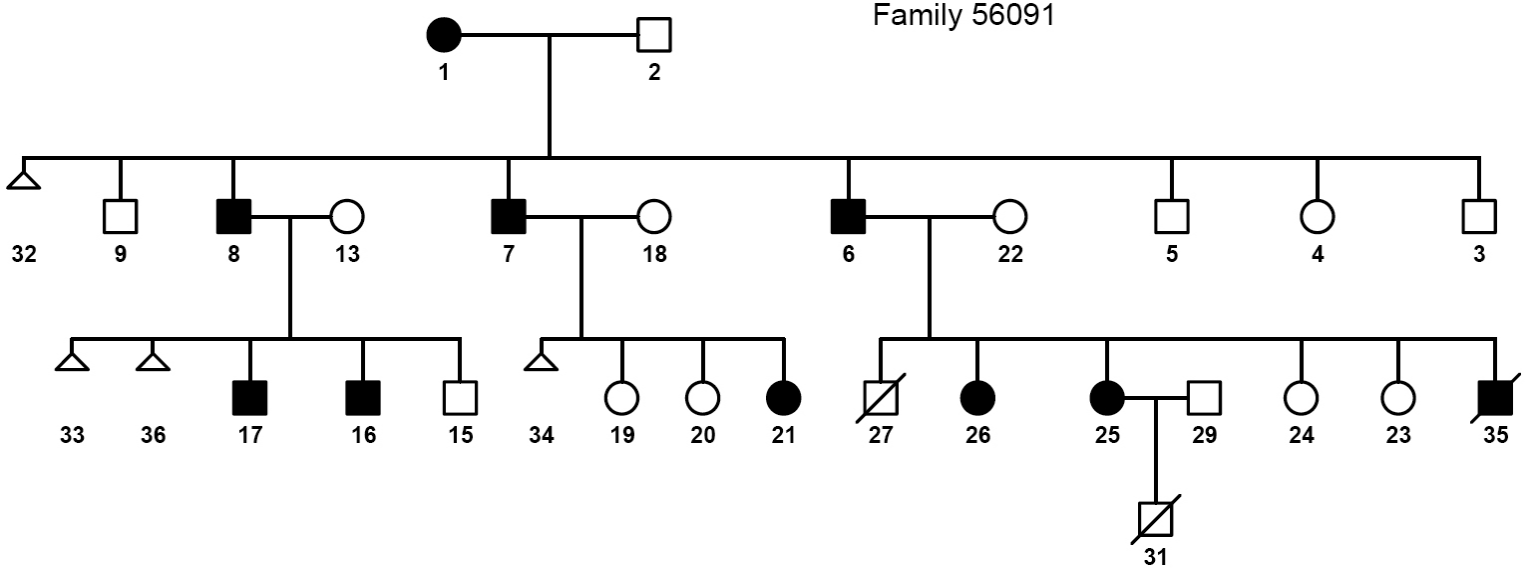
Family tree of the Libyan Jewish family. Thirteen family members underwent karyotype analysis (5609108, 5609113, 5609115, 5609116, 5609117, 5609133, 5609106, 5609122, 5609135, 5609123, 5609127, 5609125, and 5609131). Squares: males; circles: females; filled symbols: congenital cataract affected individuals; diagonal lines through symbols: deceased family members; triangles: miscarried embryos.

We have studied a three-generation Libyan Jewish family (56091) manifesting vertical transmission of congenital cataract (adCC; OMIM 604219) and a history of multiple spontaneous abortions and perinatal child deaths (Figure 1). Hospital records indicated that the cataracts were bilateral, presumably present at birth with only a minor effect on visual acuity. Karyotype results from one miscarried 11-week-old fetus (5609133) and two malformed newborns (5609127 and 5609131) with multiple congenital anomalies showed the same unbalanced translocation 46,XY, −5,+der(5)t(3:5)(p22:p15.1).

**Figure 3:**
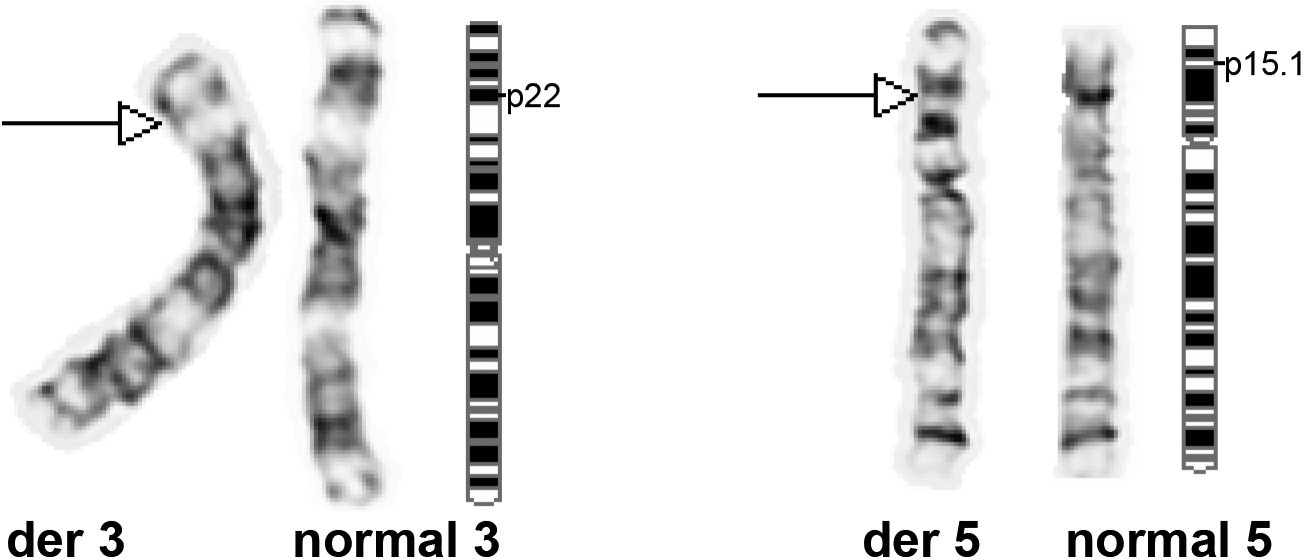
G-banded partial karyotype and ideogram. The reciprocal translocation between chromosomes 3 and 5 is illustrated. Complete karyotype notation for the rearranged chromosomes is t(3:5)(p22:p15.1). The same chromosomal translocation was found in the following congenital cataract family members: 5609108, 5609116, 5609117, 5609106, 5609125, and 5609135.

In an attempt to inquire whether the family phenotypes are related to a chromosomal rearrangement, we recruited 10 additional family members for cytogenetic and ophthalmic examinations. The study protocol was approved by the Sheba Medical Center Helsinki Committee and all participants gave informed consent.

Participants underwent a detailed ophthalmologic examination, which included slit lamp biomicroscopy and photography of the cataract lenses (when possible), at Assaf Harofeh Genetic Eye Clinic, Zerifin, Israel.

**Figure 2:**
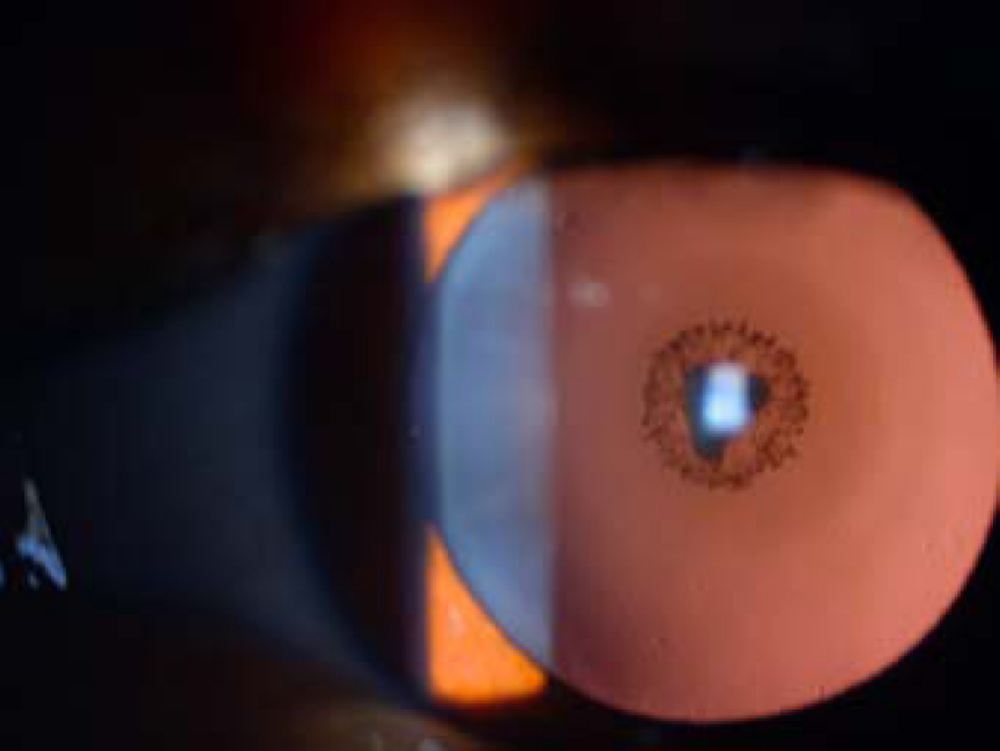
Slit lamp retro-illumination photography from an individual carrying the balanced translocation at eight years of age. The cataract is confined to the embryonic lens nucleus with dense sutural opacity surrounded by white concentric punctate opacities.

Standard cytogenetic procedures using high resolution G-banding chromosome staining were performed to analyze peripheral blood lymphocytes, amniotic fluid cells, or cultured skin fibroblasts [11]. Twenty metaphases were counted for each individual.

## Results

The cataracts (Figure 2) were confined to the embryonic lens nucleus with dense sutural opacity surrounded by white concentric punctuate opacities. Other compartments of the lens were relatively clear. They were evident in 10 members of the family (Figure 1); six of whom (5609108, 5609116, 5609117, 5609106, 5609125, and 5609135) were karyotyped and showed the same balanced reciprocal translocation [t(3:5)(p22:p15.1)] (Figure 3). Four additional healthy siblings (5609113, 5609115, 5609122, and 5609123) had normal karyotypes. Altogether, karyotype analysis was performed in 13 family members and showed cosegregation of the cataract with the balanced translocation while the unbalanced translocation product 46,XY, −5,+der(5)t(3:5)(p22:p15.1) appeared with multiple congenital anomalies. The three unbalanced translocation carriers had a similar derivative chromosome resulting in partial trisomy 3p and partial monosomy 5p. The newborns were born spontaneously at term but failed to thrive beyond infancy because of multiple congenital anomalies, which included brain ventriculomegaly, hydronephrosis, tetralogy of Fallot, and a single umbilical artery. However, they did not have cataracts.

## Discussion

Cosegregation of the cataract and the balanced translocation in this family suggests that a gene located in or remotely influenced by regulatory sites at one of the translocation breakpoints is causally related to the cataract. Previous studies have mapped a recessive congenital cataract gene to chromosome 3p23–3p21.3 and 3p22.1–3p14.2 in both Palestinian and Lebanese Arab families, respectively [12,13]. A review of their linkage data suggests an overlapping region between polymorphic markers D3S3685 and D3S2409, which correspond to the 3p22.1–3p21.3 segment. A cataract-related gene, glutathione peroxidase (GPX1; OMIM 138320), is located at this region. It was demonstrated that GPX1 knockout mice develop focal lens opacities at an early age that progress to mature cataract after 15 months, indicating a role of GPX1 in lens antioxidant defense mechanism [14]. However, sequencing its coding region failed to reveal any disease-related mutation. Distal 5p does not harbor any known cataract loci. The unbalanced chromosomal rearrangement leads to partial trisomy 3p and partial monosomy 5p without cataract. Thus, the haploinsufficiency of an altered gene product at 3p22 may be a plausible explanation for the observed ocular phenotype in the balanced translocation carriers and the lack of cataract in the unbalanced carriers. Cloning of the translocation breakpoint may allow eventual identification of a novel cataract gene and ascertain whether the previously reported cataract loci and the present one are allelic.
